# Bis(8-hy­droxy-2-methyl­quinolinium) bis­(pyridine-2,6-dicarboxyl­ato)­nickelate(II) methanol monosolvate monohydrate

**DOI:** 10.1107/S1600536811021015

**Published:** 2011-06-11

**Authors:** Hossein Aghabozorg, Ahmad Gholizadeh, Masoud Mirzaei, Behrouz Notash, Niloofar Moshki

**Affiliations:** a Faculty of Chemistry, Islamic Azad University, North Tehran Branch, Tehran, Iran; b Department of Chemistry, School of Sciences, Ferdowsi University of Mashhad, Mashhad 917791436, Iran; c Department of Chemistry, Shahid Beheshti University, G. C., Evin, Tehran 1983963113, Iran

## Abstract

In the title compound, (C_10_H_10_NO)_2_[Ni(C_7_H_3_NO_4_)_2_]·CH_3_OH·H_2_O, the coordination geometry of the Ni^II^ atom can be described as distorted octa­hedral. In the crystal, noncovalent inter­actions play an important role in the stabilization of the structure, involving O—H⋯O, N—H⋯O and weak C—H⋯O hydrogen bonds and π–π stacking inter­actions between the pyridine rings of the pyridine-2,6-dicarboxyl­ate ligands [centroid–centroid distance = 3.7138 (15) Å] and between the 8-hy­droxy-2-methyl­quinolinium cations [centroid–centroid distances = 3.6737 (15), 3.4434 (14), 3.6743 (15), 3.7541 (16), 3.5020 (15) and 3.7947 (15) Å].

## Related literature

For general background to proton transfer compounds based on carb­oxy­lic acid derivatives, see: Aghabozorg *et al.* (2008 ▶); Eshtiagh-Hosseini, Aghabozorg *et al.* (2010 ▶); Eshtiagh-Hosseini, Alfi *et al.* (2010 ▶); Eshtiagh-Hosseini, Yousefi *et al.* (2010 ▶). For related structures, see: Aghabozorg *et al.* (2011 ▶); Pasdar *et al.* (2011 ▶).
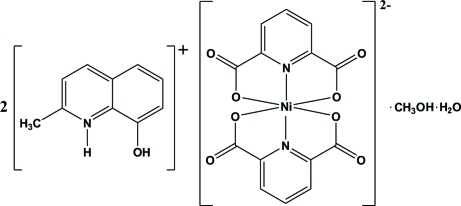


## Experimental

### 

#### Crystal data

(C_10_H_10_NO)_2_[Ni(C_7_H_3_NO_4_)_2_]·CH_4_O·H_2_O
*M* *_r_* = 759.34
Triclinic, 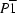

*a* = 10.100 (2) Å
*b* = 12.733 (3) Å
*c* = 14.638 (3) Å
α = 115.45 (3)°
β = 98.73 (3)°
γ = 95.89 (3)°
*V* = 1650.2 (8) Å^3^
*Z* = 2
Mo *K*α radiation
μ = 0.66 mm^−1^
*T* = 120 K
0.50 × 0.50 × 0.23 mm

#### Data collection

Stoe IPDS II diffractometer
Absorption correction: numerical (*X-SHAPE* and *X-RED32*; Stoe & Cie, 2005 ▶) *T*_min_ = 0.723, *T*_max_ = 0.856
18115 measured reflections
8795 independent reflections
7132 reflections with *I* > 2σ(*I*)
*R*_int_ = 0.047

#### Refinement

*R*[*F* ^2^ > 2σ(*F* ^2^)] = 0.047
*wR*(*F* ^2^) = 0.138
*S* = 1.05
8795 reflections
492 parameters
4 restraints
H atoms treated by a mixture of independent and constrained refinement
Δρ_max_ = 1.35 e Å^−3^
Δρ_min_ = −1.17 e Å^−3^

### 

Data collection: *X-AREA* (Stoe & Cie, 2005 ▶); cell refinement: *X-AREA*; data reduction: *X-AREA*; program(s) used to solve structure: *SHELXS97* (Sheldrick, 2008 ▶); program(s) used to refine structure: *SHELXL97* (Sheldrick, 2008 ▶); molecular graphics: *ORTEP-3* (Farrugia, 1997 ▶); software used to prepare material for publication: *WinGX* (Farrugia, 1999 ▶).

## Supplementary Material

Crystal structure: contains datablock(s) I, global. DOI: 10.1107/S1600536811021015/hy2430sup1.cif

Structure factors: contains datablock(s) I. DOI: 10.1107/S1600536811021015/hy2430Isup2.hkl

Additional supplementary materials:  crystallographic information; 3D view; checkCIF report

## Figures and Tables

**Table 1:** Hydrogen-bond geometry (Å, °)

*D*—H⋯*A*	*D*—H	H⋯*A*	*D*⋯*A*	*D*—H⋯*A*
C5—H5⋯O7^i^	0.93	2.54	3.164 (3)	124
C11—H11⋯O1^ii^	0.93	2.52	3.154 (3)	126
C15—H15*A*⋯O4^iii^	0.96	2.47	3.398 (3)	162
C17—H17⋯O6^iv^	0.93	2.27	3.155 (3)	158
C21—H21⋯O6	0.93	2.58	3.344 (3)	139
C25—H25*A*⋯O8^ii^	0.96	2.50	3.169 (3)	127
C27—H27⋯O4^v^	0.93	2.42	3.298 (3)	158
N3—H3*A*⋯O11^iii^	0.82 (3)	1.92 (3)	2.732 (3)	171 (3)
N4—H4*A*⋯O8^ii^	0.86 (3)	1.89 (3)	2.706 (3)	157 (3)
O9—H9*A*⋯O5^v^	0.82	1.75	2.574 (2)	178
O10—H10*A*⋯O2^vi^	0.82	1.76	2.562 (2)	166
O11—H11*A*⋯O3	0.87 (4)	1.83 (4)	2.699 (2)	171 (4)
O12—H12*A*⋯O7	0.82 (2)	2.05 (2)	2.852 (4)	167 (5)
O12—H12*B*⋯O4^i^	0.82 (2)	2.32 (3)	3.049 (4)	149 (4)

Symmetry codes: (i) 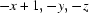; (ii) 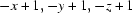; (iii) 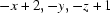; (iv) 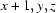; (v) 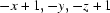; (vi) 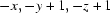.

**Fig. 1.**
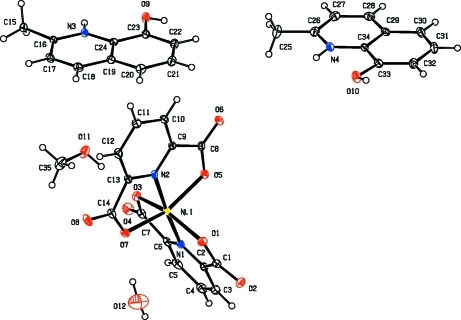
The molecular structure of the title compound, with displacement ellipsoids drawn at the 30% probability level.

**Fig. 2.**
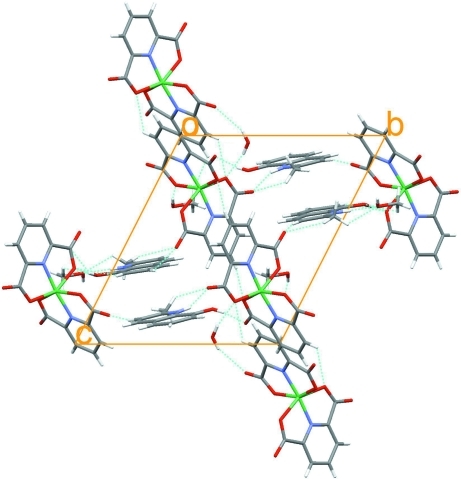
The packing diagram of the title compound. Intermolecular N—H···O, O—H···O and weak C—H···O hydrogen bonds are shown as blue dashed lines.

**Fig. 3.**
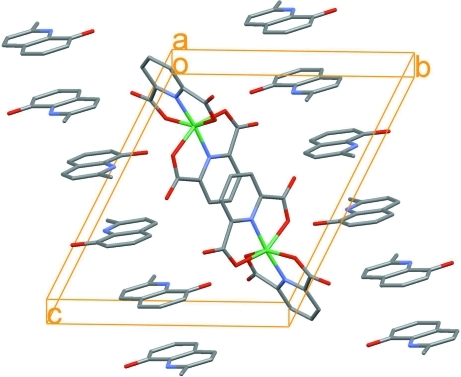
The packing diagram of the title compound, showing π–π interactions between the pyridine rings of the pydc ligands and between the 8hmqH cations. H atoms have been omitted for clarity.

## supplementary crystallographic information

### Comment

Recently, we have defined a plan to prepare water soluble proton transfer compounds as novel self-assembled systems that can function as suitable ligands in the synthesis of metal complexes. In this regard, we have reported cases in which proton transfers from pyridine-2,6-dicarboxylic acid (pydcH_2_) to different amine base ligands (Eshtiagh-Hosseini, Aghabozorg *et al.*, 2010; Eshtiagh-Hosseini, Alfi *et al.*, 2010; Eshtiagh-Hosseini, Yousefi *et al.*, 2010). This research plan has resulted in the formation of some novel proton transfer compounds based on carboxylic acid derivatives. For more details and related literature see our review article (Aghabozorg *et al.*, 2008).

We have recently reported an isostructural Cu(II) compound with formula (8hmqH)_2_[Cu(pydc)_2_].CH_3_OH.H_2_O (8hmq = 8-hydroxy-2-methylquinoline) (Aghabozorg *et al.*, 2011) and a related Ni(II) compound (Pasdar *et al.*, 2011). The molecular structure of the title compound is presented in Fig. 1. The Ni^II^ atom is six-coordinated by two pydc ligands. As it can be seen, atoms N1 and N2 of the two pydc ligands occupy the axial positions, while atoms O1, O3, O5, and O7 form the equatorial plane, with Ni—O distances ranging from 2.1247 (16) to 2.1449 (16) Å. The N1—Ni1—N2 angle [173.76 (7)°] deviates from linearity. Therefore, the geometry of the resulting NiN_2_O_4_ coordination can be described as distorted octahedral. In the crystal structure, non-covalent interactions play an important role in the stabilization of the structure, involving O—H···O, N—H···O and weak C—H···O hydrogen bonds and π–π stacking interactions between the pyridine rings of the pydc ligands [centroid–centroid distance = 3.7138 (15) Å] and between the 8hmqH cations [centroid–centroid distances = 3.6737 (15), 3.4434 (14), 3.6743 (15), 3.7541 (16), 3.5020 (15) and 3.7947 (15) Å].

### Experimental

8-Hydroxy-2-methylquinoline (0.320 g, 2 mmol) in methanol (10 ml) and 2,6-pyridine dicarboxylic acid (0.170 g, 1 mmol) in methanol (10 ml) were mixed and stirred until a clear solution was obtained. A solution of Ni(NO_3_)_2_.6H_2_O (0.145 g, 0.5 mmol) in methanol (5 ml) was added to the mixture and stirred for 30 min. Crystals of the title compound suitable for X-ray analysis were obtained by slow evaporation after two weeks.

### Refinement

H atoms bonded to N atoms and methanol O atom were found in a difference Fourier map and refined isotropically, with a restraint of N4—H4 = 0.86 (3) Å. The water H atoms were found in a difference Fourier map and refined with distance restraints of O—H = 0.82 (2) and H···H = 1.7 (4) Å and with a fixed *U*_iso_(H). H atoms bonded to C atoms and hydroxyl O atoms were positioned geometrically and refined as riding atoms, with C—H = 0.93 (aromatic) and 0.96 (methyl) and O—H = 0.82 Å and with *U*_iso_(H) = 1.2(1.5 for methyl and hydroxyl)*U*_eq_(C,O). The highest residual electron density was found at 0.83 Å from Ni1 atom and the deepest hole at 0.56 Å from O12 atom.

### Crystal data

**Table:**

(C_10_H_10_NO)_2_[Ni(C_7_H_3_NO_4_)_2_]·CH_4_O·H_2_O	*Z* = 2
*M**_r_* = 759.34	*F*(000) = 788
Triclinic, *P*1	*D*_x_ = 1.528 Mg m^−^^3^
Hall symbol: -P 1	Mo *K*α radiation, λ = 0.71073 Å
*a* = 10.100 (2) Å	Cell parameters from 8795 reflections
*b* = 12.733 (3) Å	θ = 2.1–29.1°
*c* = 14.638 (3) Å	µ = 0.66 mm^−^^1^
α = 115.45 (3)°	*T* = 120 K
β = 98.73 (3)°	Block, green
γ = 95.89 (3)°	0.50 × 0.50 × 0.23 mm
*V* = 1650.2 (8) Å^3^	

### Data collection

**Table:**

Stoe IPDS II diffractometer	8795 independent reflections
Radiation source: fine-focus sealed tube	7132 reflections with *I* > 2σ(*I*)
graphite	*R*_int_ = 0.047
ω scans	θ_max_ = 29.1°, θ_min_ = 2.1°
Absorption correction: numerical (*X-SHAPE* and *X-RED32*; Stoe & Cie, 2005)	*h* = −13→13
*T*_min_ = 0.723, *T*_max_ = 0.856	*k* = −17→17
18115 measured reflections	*l* = −20→20

### Refinement

**Table:**

Refinement on *F*^2^	Primary atom site location: structure-invariant direct methods
Least-squares matrix: full	Secondary atom site location: difference Fourier map
*R*[*F*^2^ > 2σ(*F*^2^)] = 0.047	Hydrogen site location: inferred from neighbouring sites
*wR*(*F*^2^) = 0.138	H atoms treated by a mixture of independent and constrained refinement
*S* = 1.05	*w* = 1/[σ^2^(*F*_o_^2^) + (0.095*P*)^2^] where *P* = (*F*_o_^2^ + 2*F*_c_^2^)/3
8795 reflections	(Δ/σ)_max_ < 0.001
492 parameters	Δρ_max_ = 1.35 e Å^−^^3^
4 restraints	Δρ_min_ = −1.17 e Å^−^^3^

### Fractional atomic coordinates and isotropic or equivalent isotropic displacement parameters (Å^2^)

**Table:**

	*x*	*y*	*z*	*U*_iso_*/*U*_eq_	
Ni1	0.48763 (3)	0.22447 (2)	0.253563 (19)	0.01440 (9)	
O1	0.31109 (16)	0.28626 (14)	0.21116 (11)	0.0203 (3)	
O2	0.10747 (16)	0.21828 (15)	0.10010 (13)	0.0251 (3)	
O3	0.61797 (15)	0.09757 (14)	0.23722 (12)	0.0213 (3)	
O4	0.62645 (19)	−0.09259 (16)	0.13845 (14)	0.0307 (4)	
O5	0.39775 (15)	0.19150 (14)	0.36350 (12)	0.0199 (3)	
O6	0.42156 (16)	0.25518 (16)	0.53527 (13)	0.0253 (3)	
O7	0.61440 (16)	0.32453 (13)	0.20438 (11)	0.0198 (3)	
O8	0.77752 (18)	0.48837 (15)	0.26964 (13)	0.0286 (4)	
O9	0.83619 (15)	−0.06433 (14)	0.67019 (13)	0.0220 (3)	
H9A	0.7626	−0.1060	0.6597	0.033*	
O10	−0.02135 (18)	0.58541 (16)	0.83206 (14)	0.0277 (4)	
H10A	−0.0602	0.6421	0.8462	0.042*	
O11	0.87763 (18)	0.13711 (17)	0.34025 (16)	0.0314 (4)	
O12	0.4661 (4)	0.3460 (3)	0.0331 (3)	0.0818 (10)	
N1	0.38462 (18)	0.08308 (15)	0.12842 (13)	0.0163 (3)	
N2	0.58956 (17)	0.35510 (15)	0.38661 (13)	0.0148 (3)	
N3	1.04662 (17)	0.04876 (16)	0.63184 (13)	0.0158 (3)	
N4	0.11624 (18)	0.42168 (16)	0.84640 (14)	0.0188 (3)	
C1	0.2237 (2)	0.20798 (19)	0.13492 (16)	0.0186 (4)	
C2	0.2634 (2)	0.08790 (19)	0.08134 (15)	0.0178 (4)	
C3	0.1854 (2)	−0.0107 (2)	−0.00518 (17)	0.0254 (5)	
H3	0.1011	−0.0070	−0.0382	0.030*	
C4	0.2365 (3)	−0.1150 (2)	−0.04118 (17)	0.0275 (5)	
H4	0.1863	−0.1823	−0.0990	0.033*	
C5	0.3623 (3)	−0.11857 (19)	0.00914 (17)	0.0247 (5)	
H5	0.3977	−0.1878	−0.0143	0.030*	
C6	0.4345 (2)	−0.01672 (18)	0.09532 (16)	0.0179 (4)	
C7	0.5721 (2)	−0.00531 (19)	0.16093 (17)	0.0207 (4)	
C8	0.4523 (2)	0.26155 (19)	0.45925 (16)	0.0177 (4)	
C9	0.5642 (2)	0.36054 (18)	0.47561 (15)	0.0156 (4)	
C10	0.6345 (2)	0.45077 (19)	0.57041 (16)	0.0187 (4)	
H10	0.6156	0.4548	0.6319	0.022*	
C11	0.7344 (2)	0.5355 (2)	0.57129 (16)	0.0219 (4)	
H11	0.7840	0.5967	0.6339	0.026*	
C12	0.7593 (2)	0.5279 (2)	0.47794 (17)	0.0222 (4)	
H12	0.8257	0.5838	0.4773	0.027*	
C13	0.6835 (2)	0.43572 (18)	0.38596 (15)	0.0169 (4)	
C14	0.6943 (2)	0.41509 (19)	0.27724 (16)	0.0195 (4)	
C15	1.2834 (2)	0.0523 (2)	0.61385 (17)	0.0207 (4)	
H15A	1.3271	0.0745	0.6842	0.031*	
H15B	1.3431	0.0846	0.5832	0.031*	
H15C	1.2632	−0.0324	0.5752	0.031*	
C16	1.1540 (2)	0.09952 (19)	0.61216 (15)	0.0171 (4)	
C17	1.1436 (2)	0.1982 (2)	0.59303 (16)	0.0211 (4)	
H17	1.2167	0.2331	0.5775	0.025*	
C18	1.0255 (2)	0.2424 (2)	0.59747 (17)	0.0216 (4)	
H18	1.0197	0.3083	0.5859	0.026*	
C19	0.9126 (2)	0.18995 (18)	0.61924 (16)	0.0182 (4)	
C20	0.7884 (2)	0.2320 (2)	0.62389 (17)	0.0226 (4)	
H20	0.7787	0.2982	0.6136	0.027*	
C21	0.6821 (2)	0.1746 (2)	0.64367 (18)	0.0233 (4)	
H21	0.6005	0.2025	0.6468	0.028*	
C22	0.6949 (2)	0.0737 (2)	0.65937 (17)	0.0216 (4)	
H22	0.6214	0.0359	0.6722	0.026*	
C23	0.8152 (2)	0.03039 (18)	0.65586 (15)	0.0176 (4)	
C24	0.9258 (2)	0.08958 (18)	0.63603 (15)	0.0166 (4)	
C25	0.3355 (2)	0.3609 (2)	0.8390 (2)	0.0311 (5)	
H25A	0.3341	0.3696	0.7769	0.047*	
H25B	0.3731	0.2925	0.8327	0.047*	
H25C	0.3906	0.4301	0.8972	0.047*	
C26	0.1934 (2)	0.3462 (2)	0.85504 (16)	0.0219 (4)	
C27	0.1390 (2)	0.2585 (2)	0.87963 (17)	0.0239 (4)	
H27	0.1915	0.2046	0.8850	0.029*	
C28	0.0084 (2)	0.2513 (2)	0.89586 (16)	0.0227 (4)	
H28	−0.0273	0.1921	0.9113	0.027*	
C29	−0.0716 (2)	0.33329 (19)	0.88931 (15)	0.0192 (4)	
C30	−0.2050 (2)	0.3338 (2)	0.90747 (18)	0.0251 (4)	
H30	−0.2452	0.2779	0.9248	0.030*	
C31	−0.2747 (2)	0.4172 (2)	0.89942 (18)	0.0259 (5)	
H31	−0.3625	0.4169	0.9116	0.031*	
C32	−0.2177 (2)	0.5032 (2)	0.87334 (17)	0.0234 (4)	
H32	−0.2679	0.5583	0.8680	0.028*	
C33	−0.0865 (2)	0.50617 (19)	0.85547 (16)	0.0199 (4)	
C34	−0.0139 (2)	0.42021 (19)	0.86331 (15)	0.0171 (4)	
C35	0.9526 (3)	0.2250 (3)	0.3241 (3)	0.0370 (6)	
H35A	0.9536	0.1951	0.2518	0.056*	
H35B	1.0444	0.2461	0.3638	0.056*	
H35C	0.9107	0.2937	0.3456	0.056*	
H3A	1.060 (3)	−0.010 (3)	0.640 (2)	0.030 (8)*	
H4A	0.154 (3)	0.468 (3)	0.825 (3)	0.040 (9)*	
H11A	0.794 (4)	0.117 (3)	0.304 (3)	0.051 (10)*	
H12A	0.516 (4)	0.350 (4)	0.085 (3)	0.076*	
H12B	0.419 (4)	0.291 (3)	−0.020 (2)	0.076*	

### Atomic displacement parameters (Å^2^)

**Table:**

	*U*^11^	*U*^22^	*U*^33^	*U*^12^	*U*^13^	*U*^23^
Ni1	0.01386 (13)	0.01242 (13)	0.01467 (13)	−0.00045 (9)	0.00241 (9)	0.00496 (10)
O1	0.0209 (7)	0.0180 (7)	0.0190 (7)	0.0041 (6)	0.0015 (6)	0.0063 (6)
O2	0.0182 (7)	0.0275 (8)	0.0287 (8)	0.0042 (6)	0.0006 (6)	0.0135 (7)
O3	0.0185 (7)	0.0215 (8)	0.0239 (7)	0.0048 (6)	0.0046 (6)	0.0101 (6)
O4	0.0376 (10)	0.0272 (9)	0.0353 (9)	0.0177 (8)	0.0153 (8)	0.0164 (8)
O5	0.0176 (7)	0.0198 (7)	0.0218 (7)	−0.0027 (6)	0.0049 (6)	0.0102 (6)
O6	0.0224 (8)	0.0343 (9)	0.0252 (8)	0.0001 (7)	0.0080 (6)	0.0192 (7)
O7	0.0233 (7)	0.0179 (7)	0.0152 (6)	−0.0015 (6)	0.0052 (6)	0.0055 (6)
O8	0.0325 (9)	0.0242 (8)	0.0258 (8)	−0.0073 (7)	0.0148 (7)	0.0085 (7)
O9	0.0155 (7)	0.0219 (8)	0.0323 (8)	−0.0004 (6)	0.0055 (6)	0.0165 (7)
O10	0.0278 (8)	0.0280 (9)	0.0395 (9)	0.0097 (7)	0.0165 (7)	0.0225 (8)
O11	0.0183 (8)	0.0322 (9)	0.0500 (11)	0.0004 (7)	0.0011 (7)	0.0273 (9)
O12	0.100 (3)	0.080 (2)	0.0591 (18)	0.031 (2)	0.0050 (18)	0.0261 (17)
N1	0.0170 (8)	0.0145 (8)	0.0168 (8)	0.0001 (6)	0.0054 (6)	0.0068 (6)
N2	0.0131 (7)	0.0141 (8)	0.0160 (7)	0.0005 (6)	0.0044 (6)	0.0056 (6)
N3	0.0151 (8)	0.0147 (8)	0.0161 (7)	−0.0007 (6)	0.0035 (6)	0.0063 (6)
N4	0.0177 (8)	0.0189 (8)	0.0175 (8)	0.0005 (7)	0.0057 (6)	0.0063 (7)
C1	0.0195 (9)	0.0204 (10)	0.0169 (9)	0.0033 (8)	0.0048 (7)	0.0092 (8)
C2	0.0170 (9)	0.0193 (10)	0.0150 (9)	−0.0006 (8)	0.0042 (7)	0.0064 (8)
C3	0.0223 (10)	0.0287 (12)	0.0183 (10)	−0.0041 (9)	0.0029 (8)	0.0067 (9)
C4	0.0319 (12)	0.0228 (11)	0.0173 (10)	−0.0079 (9)	0.0059 (9)	0.0022 (8)
C5	0.0382 (13)	0.0139 (9)	0.0206 (10)	0.0002 (9)	0.0139 (9)	0.0053 (8)
C6	0.0221 (10)	0.0154 (9)	0.0179 (9)	0.0014 (8)	0.0097 (7)	0.0078 (8)
C7	0.0232 (10)	0.0202 (10)	0.0239 (10)	0.0060 (8)	0.0114 (8)	0.0123 (8)
C8	0.0143 (9)	0.0201 (10)	0.0220 (9)	0.0024 (7)	0.0052 (7)	0.0124 (8)
C9	0.0141 (8)	0.0174 (9)	0.0174 (9)	0.0022 (7)	0.0045 (7)	0.0095 (7)
C10	0.0204 (9)	0.0204 (10)	0.0158 (9)	0.0040 (8)	0.0059 (7)	0.0080 (8)
C11	0.0224 (10)	0.0193 (10)	0.0168 (9)	−0.0014 (8)	0.0032 (8)	0.0031 (8)
C12	0.0204 (10)	0.0183 (10)	0.0219 (10)	−0.0055 (8)	0.0050 (8)	0.0054 (8)
C13	0.0173 (9)	0.0153 (9)	0.0177 (9)	0.0007 (7)	0.0068 (7)	0.0067 (7)
C14	0.0215 (10)	0.0169 (9)	0.0193 (9)	0.0012 (8)	0.0092 (8)	0.0066 (8)
C15	0.0148 (9)	0.0247 (10)	0.0211 (10)	0.0003 (8)	0.0039 (7)	0.0098 (8)
C16	0.0145 (9)	0.0200 (9)	0.0128 (8)	−0.0026 (7)	0.0022 (7)	0.0053 (7)
C17	0.0221 (10)	0.0209 (10)	0.0189 (9)	−0.0035 (8)	0.0053 (8)	0.0090 (8)
C18	0.0255 (11)	0.0192 (10)	0.0212 (10)	0.0001 (8)	0.0044 (8)	0.0113 (8)
C19	0.0189 (9)	0.0171 (9)	0.0164 (9)	−0.0006 (8)	0.0029 (7)	0.0069 (7)
C20	0.0243 (10)	0.0202 (10)	0.0233 (10)	0.0044 (8)	0.0043 (8)	0.0101 (8)
C21	0.0182 (10)	0.0247 (11)	0.0254 (10)	0.0050 (8)	0.0045 (8)	0.0098 (9)
C22	0.0165 (9)	0.0227 (10)	0.0240 (10)	0.0003 (8)	0.0046 (8)	0.0099 (8)
C23	0.0167 (9)	0.0169 (9)	0.0167 (9)	−0.0009 (7)	0.0025 (7)	0.0067 (7)
C24	0.0160 (9)	0.0165 (9)	0.0138 (8)	−0.0010 (7)	0.0018 (7)	0.0050 (7)
C25	0.0181 (10)	0.0263 (12)	0.0396 (13)	0.0032 (9)	0.0066 (9)	0.0067 (10)
C26	0.0198 (10)	0.0194 (10)	0.0190 (9)	0.0024 (8)	0.0029 (8)	0.0026 (8)
C27	0.0255 (11)	0.0199 (10)	0.0220 (10)	0.0053 (8)	0.0009 (8)	0.0065 (8)
C28	0.0294 (11)	0.0194 (10)	0.0181 (9)	0.0003 (8)	0.0036 (8)	0.0088 (8)
C29	0.0214 (10)	0.0198 (10)	0.0135 (8)	−0.0004 (8)	0.0041 (7)	0.0056 (7)
C30	0.0243 (11)	0.0264 (11)	0.0235 (10)	−0.0025 (9)	0.0100 (8)	0.0103 (9)
C31	0.0178 (10)	0.0286 (12)	0.0270 (11)	0.0013 (9)	0.0099 (8)	0.0076 (9)
C32	0.0205 (10)	0.0235 (11)	0.0242 (10)	0.0058 (8)	0.0070 (8)	0.0081 (9)
C33	0.0208 (10)	0.0197 (10)	0.0187 (9)	0.0020 (8)	0.0051 (8)	0.0083 (8)
C34	0.0165 (9)	0.0190 (9)	0.0138 (8)	0.0015 (7)	0.0050 (7)	0.0057 (7)
C35	0.0280 (12)	0.0318 (13)	0.0547 (17)	−0.0047 (10)	−0.0021 (11)	0.0284 (13)

### Geometric parameters (Å, °)

**Table:**

Ni1—N2	1.969 (2)	C11—C12	1.392 (3)
Ni1—N1	1.970 (2)	C11—H11	0.9300
Ni1—O7	2.1247 (16)	C12—C13	1.386 (3)
Ni1—O5	2.1298 (16)	C12—H12	0.9300
Ni1—O1	2.1343 (16)	C13—C14	1.521 (3)
Ni1—O3	2.1449 (16)	C15—C16	1.495 (3)
O1—C1	1.258 (3)	C15—H15A	0.9600
O2—C1	1.250 (3)	C15—H15B	0.9600
O3—C7	1.280 (3)	C15—H15C	0.9600
O4—C7	1.229 (3)	C16—C17	1.410 (3)
O5—C8	1.286 (3)	C17—C18	1.369 (3)
O6—C8	1.232 (3)	C17—H17	0.9300
O7—C14	1.269 (3)	C18—C19	1.409 (3)
O8—C14	1.243 (3)	C18—H18	0.9300
O9—C23	1.342 (3)	C19—C20	1.413 (3)
O9—H9A	0.8200	C19—C24	1.417 (3)
O10—C33	1.341 (3)	C20—C21	1.373 (3)
O10—H10A	0.8200	C20—H20	0.9300
O11—C35	1.411 (3)	C21—C22	1.413 (3)
O11—H11A	0.87 (4)	C21—H21	0.9300
O12—H12A	0.82 (2)	C22—C23	1.384 (3)
O12—H12B	0.82 (2)	C22—H22	0.9300
N1—C2	1.332 (3)	C23—C24	1.419 (3)
N1—C6	1.334 (3)	C25—C26	1.495 (3)
N2—C13	1.329 (3)	C25—H25A	0.9600
N2—C9	1.340 (3)	C25—H25B	0.9600
N3—C16	1.337 (3)	C25—H25C	0.9600
N3—C24	1.374 (3)	C26—C27	1.400 (3)
N3—H3A	0.82 (3)	C27—C28	1.376 (3)
N4—C26	1.333 (3)	C27—H27	0.9300
N4—C34	1.374 (3)	C28—C29	1.410 (3)
N4—H4A	0.86 (3)	C28—H28	0.9300
C1—C2	1.518 (3)	C29—C30	1.412 (3)
C2—C3	1.389 (3)	C29—C34	1.416 (3)
C3—C4	1.389 (4)	C30—C31	1.368 (4)
C3—H3	0.9300	C30—H30	0.9300
C4—C5	1.384 (4)	C31—C32	1.405 (3)
C4—H4	0.9300	C31—H31	0.9300
C5—C6	1.388 (3)	C32—C33	1.389 (3)
C5—H5	0.9300	C32—H32	0.9300
C6—C7	1.519 (3)	C33—C34	1.413 (3)
C8—C9	1.513 (3)	C35—H35A	0.9600
C9—C10	1.383 (3)	C35—H35B	0.9600
C10—C11	1.394 (3)	C35—H35C	0.9600
C10—H10	0.9300		
			
N2—Ni1—N1	173.76 (7)	O8—C14—C13	117.26 (19)
N2—Ni1—O7	78.07 (7)	O7—C14—C13	115.06 (18)
N1—Ni1—O7	107.32 (7)	C16—C15—H15A	109.5
N2—Ni1—O5	77.35 (7)	C16—C15—H15B	109.5
N1—Ni1—O5	97.45 (7)	H15A—C15—H15B	109.5
O7—Ni1—O5	155.11 (6)	C16—C15—H15C	109.5
N2—Ni1—O1	105.25 (7)	H15A—C15—H15C	109.5
N1—Ni1—O1	77.98 (7)	H15B—C15—H15C	109.5
O7—Ni1—O1	92.46 (6)	N3—C16—C17	119.24 (19)
O5—Ni1—O1	90.26 (6)	N3—C16—C15	119.15 (19)
N2—Ni1—O3	99.28 (7)	C17—C16—C15	121.58 (19)
N1—Ni1—O3	77.43 (7)	C18—C17—C16	119.7 (2)
O7—Ni1—O3	94.27 (6)	C18—C17—H17	120.1
O5—Ni1—O3	93.46 (6)	C16—C17—H17	120.1
O1—Ni1—O3	155.41 (6)	C17—C18—C19	121.2 (2)
C1—O1—Ni1	114.11 (14)	C17—C18—H18	119.4
C7—O3—Ni1	115.21 (13)	C19—C18—H18	119.4
C8—O5—Ni1	115.57 (13)	C18—C19—C20	123.2 (2)
C14—O7—Ni1	114.82 (13)	C18—C19—C24	117.50 (19)
C23—O9—H9A	109.5	C20—C19—C24	119.32 (19)
C33—O10—H10A	109.5	C21—C20—C19	119.7 (2)
C35—O11—H11A	111 (3)	C21—C20—H20	120.2
H12A—O12—H12B	134 (4)	C19—C20—H20	120.2
C2—N1—C6	121.35 (19)	C20—C21—C22	121.0 (2)
C2—N1—Ni1	119.01 (14)	C20—C21—H21	119.5
C6—N1—Ni1	119.57 (14)	C22—C21—H21	119.5
C13—N2—C9	121.36 (18)	C23—C22—C21	120.9 (2)
C13—N2—Ni1	118.90 (14)	C23—C22—H22	119.5
C9—N2—Ni1	119.74 (14)	C21—C22—H22	119.5
C16—N3—C24	122.90 (18)	O9—C23—C22	124.84 (19)
C16—N3—H3A	113 (2)	O9—C23—C24	116.69 (18)
C24—N3—H3A	124 (2)	C22—C23—C24	118.46 (19)
C26—N4—C34	123.3 (2)	N3—C24—C19	119.42 (18)
C26—N4—H4A	113 (2)	N3—C24—C23	119.95 (19)
C34—N4—H4A	123 (2)	C19—C24—C23	120.62 (19)
O2—C1—O1	126.9 (2)	C26—C25—H25A	109.5
O2—C1—C2	116.94 (19)	C26—C25—H25B	109.5
O1—C1—C2	116.19 (18)	H25A—C25—H25B	109.5
N1—C2—C3	121.0 (2)	C26—C25—H25C	109.5
N1—C2—C1	112.51 (18)	H25A—C25—H25C	109.5
C3—C2—C1	126.51 (19)	H25B—C25—H25C	109.5
C2—C3—C4	118.4 (2)	N4—C26—C27	119.0 (2)
C2—C3—H3	120.8	N4—C26—C25	117.8 (2)
C4—C3—H3	120.8	C27—C26—C25	123.2 (2)
C5—C4—C3	119.9 (2)	C28—C27—C26	120.4 (2)
C5—C4—H4	120.1	C28—C27—H27	119.8
C3—C4—H4	120.1	C26—C27—H27	119.8
C4—C5—C6	118.6 (2)	C27—C28—C29	120.4 (2)
C4—C5—H5	120.7	C27—C28—H28	119.8
C6—C5—H5	120.7	C29—C28—H28	119.8
N1—C6—C5	120.8 (2)	C28—C29—C30	123.9 (2)
N1—C6—C7	113.46 (18)	C28—C29—C34	117.7 (2)
C5—C6—C7	125.7 (2)	C30—C29—C34	118.4 (2)
O4—C7—O3	126.7 (2)	C31—C30—C29	119.6 (2)
O4—C7—C6	119.0 (2)	C31—C30—H30	120.2
O3—C7—C6	114.29 (18)	C29—C30—H30	120.2
O6—C8—O5	126.6 (2)	C30—C31—C32	122.0 (2)
O6—C8—C9	119.24 (19)	C30—C31—H31	119.0
O5—C8—C9	114.17 (18)	C32—C31—H31	119.0
N2—C9—C10	121.21 (19)	C33—C32—C31	120.2 (2)
N2—C9—C8	112.99 (18)	C33—C32—H32	119.9
C10—C9—C8	125.79 (18)	C31—C32—H32	119.9
C9—C10—C11	118.25 (19)	O10—C33—C32	125.2 (2)
C9—C10—H10	120.9	O10—C33—C34	116.59 (19)
C11—C10—H10	120.9	C32—C33—C34	118.2 (2)
C12—C11—C10	119.6 (2)	N4—C34—C33	119.24 (19)
C12—C11—H11	120.2	N4—C34—C29	119.19 (19)
C10—C11—H11	120.2	C33—C34—C29	121.57 (19)
C13—C12—C11	118.8 (2)	O11—C35—H35A	109.5
C13—C12—H12	120.6	O11—C35—H35B	109.5
C11—C12—H12	120.6	H35A—C35—H35B	109.5
N2—C13—C12	120.76 (19)	O11—C35—H35C	109.5
N2—C13—C14	113.11 (18)	H35A—C35—H35C	109.5
C12—C13—C14	126.12 (19)	H35B—C35—H35C	109.5
O8—C14—O7	127.7 (2)		
			
N2—Ni1—O1—C1	−170.54 (14)	Ni1—N2—C9—C8	−1.5 (2)
N1—Ni1—O1—C1	3.97 (15)	O6—C8—C9—N2	178.34 (18)
O7—Ni1—O1—C1	111.16 (15)	O5—C8—C9—N2	−2.0 (2)
O5—Ni1—O1—C1	−93.59 (15)	O6—C8—C9—C10	−2.9 (3)
O3—Ni1—O1—C1	5.3 (2)	O5—C8—C9—C10	176.70 (19)
N2—Ni1—O3—C7	173.19 (15)	N2—C9—C10—C11	−0.9 (3)
N1—Ni1—O3—C7	−1.41 (15)	C8—C9—C10—C11	−179.59 (19)
O7—Ni1—O3—C7	−108.22 (15)	C9—C10—C11—C12	0.7 (3)
O5—Ni1—O3—C7	95.46 (15)	C10—C11—C12—C13	0.1 (3)
O1—Ni1—O3—C7	−2.7 (2)	C9—N2—C13—C12	0.5 (3)
N2—Ni1—O5—C8	−4.03 (14)	Ni1—N2—C13—C12	−178.84 (16)
N1—Ni1—O5—C8	172.48 (14)	C9—N2—C13—C14	−178.75 (17)
O7—Ni1—O5—C8	−13.2 (2)	Ni1—N2—C13—C14	1.9 (2)
O1—Ni1—O5—C8	−109.61 (15)	C11—C12—C13—N2	−0.7 (3)
O3—Ni1—O5—C8	94.72 (15)	C11—C12—C13—C14	178.5 (2)
N2—Ni1—O7—C14	1.19 (15)	Ni1—O7—C14—O8	−179.45 (19)
N1—Ni1—O7—C14	−175.57 (14)	Ni1—O7—C14—C13	−0.5 (2)
O5—Ni1—O7—C14	10.3 (2)	N2—C13—C14—O8	178.21 (19)
O1—Ni1—O7—C14	106.26 (15)	C12—C13—C14—O8	−1.0 (3)
O3—Ni1—O7—C14	−97.41 (15)	N2—C13—C14—O7	−0.8 (3)
O7—Ni1—N1—C2	−90.88 (16)	C12—C13—C14—O7	180.0 (2)
O5—Ni1—N1—C2	86.61 (16)	C24—N3—C16—C17	−1.1 (3)
O1—Ni1—N1—C2	−2.05 (15)	C24—N3—C16—C15	177.27 (18)
O3—Ni1—N1—C2	178.52 (16)	N3—C16—C17—C18	1.7 (3)
O7—Ni1—N1—C6	92.11 (16)	C15—C16—C17—C18	−176.62 (19)
O5—Ni1—N1—C6	−90.39 (16)	C16—C17—C18—C19	−1.1 (3)
O1—Ni1—N1—C6	−179.06 (16)	C17—C18—C19—C20	−179.5 (2)
O3—Ni1—N1—C6	1.51 (15)	C17—C18—C19—C24	−0.2 (3)
O7—Ni1—N2—C13	−1.72 (15)	C18—C19—C20—C21	178.8 (2)
O5—Ni1—N2—C13	−177.78 (16)	C24—C19—C20—C21	−0.6 (3)
O1—Ni1—N2—C13	−91.02 (15)	C19—C20—C21—C22	−0.1 (3)
O3—Ni1—N2—C13	90.73 (15)	C20—C21—C22—C23	0.4 (3)
O7—Ni1—N2—C9	178.94 (16)	C21—C22—C23—O9	179.8 (2)
O5—Ni1—N2—C9	2.87 (14)	C21—C22—C23—C24	0.0 (3)
O1—Ni1—N2—C9	89.63 (15)	C16—N3—C24—C19	−0.2 (3)
O3—Ni1—N2—C9	−88.62 (15)	C16—N3—C24—C23	179.11 (18)
Ni1—O1—C1—O2	173.66 (18)	C18—C19—C24—N3	0.8 (3)
Ni1—O1—C1—C2	−5.0 (2)	C20—C19—C24—N3	−179.81 (18)
C6—N1—C2—C3	−1.1 (3)	C18—C19—C24—C23	−178.48 (18)
Ni1—N1—C2—C3	−178.01 (16)	C20—C19—C24—C23	0.9 (3)
C6—N1—C2—C1	177.15 (17)	O9—C23—C24—N3	0.3 (3)
Ni1—N1—C2—C1	0.2 (2)	C22—C23—C24—N3	−179.88 (19)
O2—C1—C2—N1	−175.41 (18)	O9—C23—C24—C19	179.56 (18)
O1—C1—C2—N1	3.4 (3)	C22—C23—C24—C19	−0.6 (3)
O2—C1—C2—C3	2.7 (3)	C34—N4—C26—C27	1.9 (3)
O1—C1—C2—C3	−178.5 (2)	C34—N4—C26—C25	−177.4 (2)
N1—C2—C3—C4	0.4 (3)	N4—C26—C27—C28	−0.8 (3)
C1—C2—C3—C4	−177.5 (2)	C25—C26—C27—C28	178.4 (2)
C2—C3—C4—C5	0.0 (3)	C26—C27—C28—C29	−0.9 (3)
C3—C4—C5—C6	0.2 (3)	C27—C28—C29—C30	−178.3 (2)
C2—N1—C6—C5	1.2 (3)	C27—C28—C29—C34	1.4 (3)
Ni1—N1—C6—C5	178.16 (15)	C28—C29—C30—C31	179.9 (2)
C2—N1—C6—C7	−178.34 (18)	C34—C29—C30—C31	0.1 (3)
Ni1—N1—C6—C7	−1.4 (2)	C29—C30—C31—C32	0.1 (4)
C4—C5—C6—N1	−0.8 (3)	C30—C31—C32—C33	−0.4 (4)
C4—C5—C6—C7	178.7 (2)	C31—C32—C33—O10	−178.7 (2)
Ni1—O3—C7—O4	−177.46 (19)	C31—C32—C33—C34	0.6 (3)
Ni1—O3—C7—C6	1.1 (2)	C26—N4—C34—C33	178.0 (2)
N1—C6—C7—O4	178.78 (19)	C26—N4—C34—C29	−1.3 (3)
C5—C6—C7—O4	−0.8 (3)	O10—C33—C34—N4	−0.4 (3)
N1—C6—C7—O3	0.1 (3)	C32—C33—C34—N4	−179.72 (19)
C5—C6—C7—O3	−179.4 (2)	O10—C33—C34—C29	178.94 (19)
Ni1—O5—C8—O6	−176.08 (17)	C32—C33—C34—C29	−0.4 (3)
Ni1—O5—C8—C9	4.3 (2)	C28—C29—C34—N4	−0.4 (3)
C13—N2—C9—C10	0.3 (3)	C30—C29—C34—N4	179.36 (19)
Ni1—N2—C9—C10	179.66 (14)	C28—C29—C34—C33	−179.72 (19)
C13—N2—C9—C8	179.14 (17)	C30—C29—C34—C33	0.1 (3)

### Hydrogen-bond geometry (Å, °)

**Table:**

*D*—H···*A*	*D*—H	H···*A*	*D*···*A*	*D*—H···*A*
C5—H5···O7^i^	0.93	2.54	3.164 (3)	124.
C11—H11···O1^ii^	0.93	2.52	3.154 (3)	126.
C15—H15A···O4^iii^	0.96	2.47	3.398 (3)	162.
C17—H17···O6^iv^	0.93	2.27	3.155 (3)	158.
C21—H21···O6	0.93	2.58	3.344 (3)	139.
C25—H25A···O8^ii^	0.96	2.50	3.169 (3)	127.
C27—H27···O4^v^	0.93	2.42	3.298 (3)	158.
N3—H3A···O11^iii^	0.82 (3)	1.92 (3)	2.732 (3)	171 (3)
N4—H4A···O8^ii^	0.86 (3)	1.89 (3)	2.706 (3)	157 (3)
O9—H9A···O5^v^	0.82	1.75	2.574 (2)	178.
O10—H10A···O2^vi^	0.82	1.76	2.562 (2)	166.
O11—H11A···O3	0.87 (4)	1.83 (4)	2.699 (2)	171 (4)
O12—H12A···O7	0.82 (2)	2.05 (2)	2.852 (4)	167 (5)
O12—H12B···O4^i^	0.82 (2)	2.32 (3)	3.049 (4)	149 (4)

Symmetry codes: (i) −*x*+1, −*y*, −*z*; (ii) −*x*+1, −*y*+1, −*z*+1; (iii) −*x*+2, −*y*, −*z*+1; (iv) *x*+1, *y*, *z*; (v) −*x*+1, −*y*, −*z*+1; (vi) −*x*, −*y*+1, −*z*+1.
